# Performance of dRAST on Prospective Clinical Blood Culture Samples in a Simulated Clinical Setting and on Multidrug-Resistant Bacteria

**DOI:** 10.1128/spectrum.02107-21

**Published:** 2022-03-02

**Authors:** Alicia Y. W. Wong, Alexander T. A. Johnsson, Volkan Özenci

**Affiliations:** a Division of Clinical Microbiology, Department of Laboratory Medicine, Karolinska Institutet, Huddinge, Stockholm, Sweden; b Department of Clinical Microbiology, Karolinska University Hospital, Huddinge, Stockholm, Sweden; Montefiore Medical Center and Albert Einstein College of Medicine

**Keywords:** automated antimicrobial susceptibility testing, bacteremia, blood culture, dRAST

## Abstract

There is an utmost need for rapid antimicrobial susceptibility testing (AST) of bacteria causing bloodstream infections (BSI). The dRAST (QuantaMatrix Inc., Seoul) is a commercial method that can be performed directly from positive blood cultures. The present study aims to evaluate the performance of the dRAST on prospective clinical blood culture samples. A sample prescreening algorithm based on clinical routine was used to choose relevant clinical positive blood culture samples for testing on the dRAST. Rapid identification via short-term culture followed by matrix-assisted laser desorption ionization–time of flight mass spectrometry (MALDI-TOF MS) was used during the test run, and dRAST results were compared to European Committee on Antimicrobial Susceptibility Testing (EUCAST) disk diffusion as the reference method. The performance of the dRAST was also evaluated on selected multidrug resistant (MDR) isolates in simulated blood cultures. Using the sample pre-screening algorithm, 242 clinical blood culture samples were selected and tested on the dRAST, of which 200 (82.6%) gave valid AST tests results comprising 76 Gram-positive and 124 Gram-negative samples. AST measurements from the dRAST and disk diffusion from clinical samples had an overall agreement rate of 95.5%. When using simulated blood culture samples of 31 selected MDR isolates, the agreement between dRAST and disk diffusion was 87.2%. While the agreement rates were high, it was noted that the dRAST was not reliable for AST of certain antibiotic–bacteria combinations. In conclusion, the present study demonstrates that dRAST delivers rapid AST results from blood cultures and using a prescreening algorithm for sample selection is important in implementation of modern AST methods such as dRAST.

**IMPORTANCE** There is an utmost need for rapid antimicrobial susceptibility testing (AST) of bacteria causing bloodstream infections (BSI). The dRAST (QuantaMatrix Inc., Seoul) is a rapid AST method that can be performed directly from positive blood cultures. The dRAST gives results in 6 h compared to conventional AST methods that needs 18-20 h of incubation. The present study aims to evaluate the performance of the dRAST in a clinical setting with the use of a sample selection algorithm to reduce incompatible sample numbers. The study found that while the agreement rates between dRAST and reference AST methods were high, it was noted that the dRAST was not reliable for AST of certain antibiotic–bacteria combinations. In conclusion, the present study demonstrates that dRAST delivers rapid AST results from blood cultures and using a prescreening algorithm for sample selection is important in implementation of modern AST methods such as dRAST.

## INTRODUCTION

Bloodstream infections (BSIs) pose a significant global health care burden and in 2017 were estimated to have a case fatality rate of 12–20% (1). It is well established that patient survival from BSI is dependent on the rapid administration of effective antimicrobial treatment (2, 3). However, 18–20 h is needed to incubate disk diffusion plates according to the European Committee on Antimicrobial Susceptibility Testing (EUCAST) method before antimicrobial susceptibility testing (AST) of causative microorganisms can be assessed (4). Furthermore, the rise in rates of antimicrobial-resistant bacteria also contributes to mortality since patients infected with such pathogens are also likely to receive ineffective antibiotic treatment leading to a poorer clinical outcome, or be treated with overly broad antibiotics that can increase the risk of adverse side effects and further drive development of resistance in pathogens (5). It is thus of great interest to shorten the time required to perform AST.

Rapid AST methods have been developed in recent years to shorten the diagnosis process. This includes the EUCAST rapid AST, which is based on performing the standard disk diffusion test directly using positive blood culture, which are then incubated for 4–8 h or 16–18 h (6). Although the EUCAST rapid AST is capable of providing more rapid results, the method is performed manually and is labor-intensive due to the strict timing needed for AST interpretations. Other phenotypic rapid AST methods utilizing novel technologies have also been developed to automate AST directly on positive blood culture samples, and most provide AST results in as little as 4–7 h, therefore bypassing the lengthy subculturing and manual AST setup used in conventional methods and shortening time to result (5).

The dRAST system (QuantaMatrix Inc., Seoul) is a novel rapid antimicrobial susceptibility testing (AST) system that recently achieved Conformité-Européenne *in vitro* Diagnostic (CE-IVD) approval. It is capable of giving phenotypic AST results directly from positive blood cultures or from colony isolates within 6 h, using automated microscopy to analyze bacteria growth in agar in the presence of a panel of antimicrobial agents that were designed based on either Clinical and Laboratory Standards Institute (CLSI) or EUCAST recommendations (7). Several published prospective studies evaluating the dRAST were focused on staphylococci and enterococci (8, 9), and on Gram-negative bacteria (10, 11). The reported categorical agreement (CA) between the dRAST system and various reference AST methods was over 90% (7, 8, 10, 11).

The present study aims to evaluate the performance of the dRAST on prospective clinical blood culture samples. The study was designed to simulate how the dRAST might be used in our clinical routine where the primary AST method is the EUCAST standardized disk diffusion testing (4, 12). Positive blood culture samples were selected with a prescreening algorithm to identify blood culture samples that would be compatible with the dRAST panel, and results obtained were evaluated primarily for CA. In addition, as Sweden experiences generally low levels of antibiotic resistance (13), the present study also evaluated the performance of the dRAST on simulated blood culture samples inoculated with selected multidrug resistant (MDR) clinical isolates.

## RESULTS

### Performance of a sample selection algorithm for clinical blood cultures.

**FIG 1:**
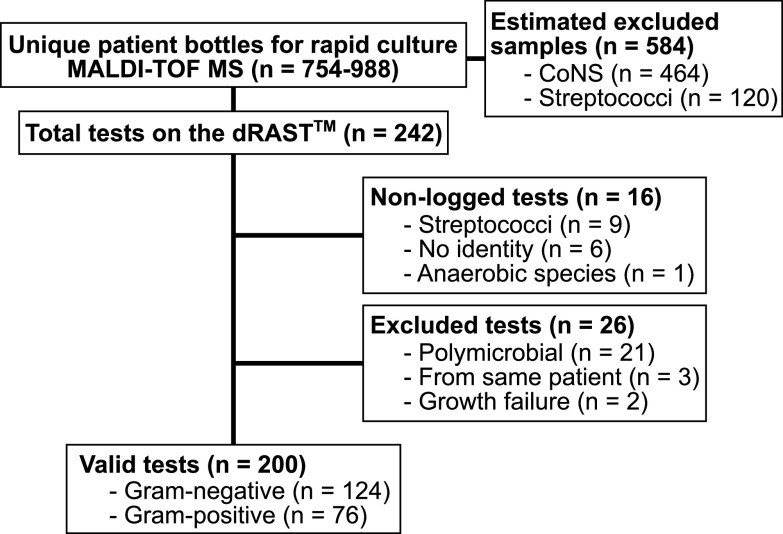
Tests run on the dRAST. Breakdown of estimated blood culture samples considered during study period, and number of valid tests versus nonlogged or excluded tests of blood culture samples that were run on the dRAST.

The clinic receives 58–76 unique patient bottles/week that are marked for rapid culture matrix-assisted laser desorption ionization–time of flight mass spectrometry (MALDI-TOF MS) (Table S1 in the supplemental material), and receives on average 9 streptococci and 36 coagulase-negative staphylococci (CoNS) isolates/week. Hence it is estimated that there were 754–988 unique patient bottles that were marked for rapid culture MALDI-TOF MS during the study period, and that the number of streptococci and CoNS isolates that would be encountered during the study period were 120 and 464, respectively (Table S1, Fig. 1). Using the sample selection algorithm, a total of 242 blood culture samples were selected from the clinical routine and tested on the dRAST. Of the 242 blood culture samples, there were 200 (82.6%) valid tests, which comprised 77 (38.5%) Gram-positive samples and 123 (61.5%) Gram-negative samples. The remaining 42 tests that were not included in the present study either were not successfully logged in the dRAST system (incompatible species such as Streptococcus spp. or anaerobic bacteria, or lack of identity with the routine method) or were eventually excluded from analysis because they were determined to be polymicrobial samples or multiple samples taken from the same patient, or because the isolate failed to grow during incubation in the dRAST (Fig. 1). The two samples that were reported as growth failures in the dRAST were one Gram-positive isolate (Staphylococcus epidermidis) and one Gram-negative non-enterobacteriaceae isolate that could not be further identified by routine methods.

### Species distribution in valid clinical blood culture samples.

**FIG 2:**
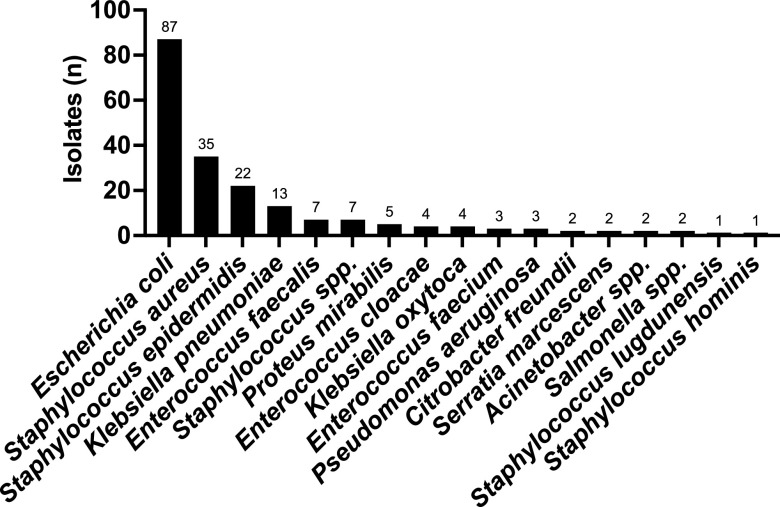
Species distribution of valid dRAST tests performed on clinical blood culture samples.

From the 200 valid monomicrobial clinical samples run on the dRAST system, the majority isolated Gram-positive and Gram-negative species were S. aureus (35/200, 17.5%) and E. coli (87/200, 43.5%), respectively. The detailed distribution of species encountered in valid clinical blood culture samples is presented in Fig. 2.

### Performance of the dRAST on prospective clinical blood culture samples.

The majority of isolates from prospective clinical blood culture samples were determined to be susceptible strains by disk diffusion (Table S2 and S3). A total of 1,682 AST measurements comparing the dRAST with disk diffusion were done on the 200 valid clinical samples, and there was a 95.5% overall agreement rate in the results obtained by the dRAST with disk diffusion. Of the 473 (28.1%) Gram-positive AST measurements, discrepant results obtained were 14 very major error (VME), 5 major error (ME), and 7 minor error (mE), giving an overall agreement rate for Gram-positive AST measurements of 94.5% (Table 1). For vancomycin, 10 tests between the dRAST and disk diffusion could be done. These tests were performed on seven E. faecalis and three E. faecium, and all results between the dRAST and disk diffusion were in agreement. The AST results where discrepancies were encountered for Gram-positive bacteria include clindamycin, erythromycin, fusidic acid, gentamicin, rifampin, and inducible clindamycin resistance (Table 1, Table S4).

**TABLE 1:** dRAST versus reference method for Gram-positive bacteria from prospective clinical blood culture samples^*a*^

Antibiotic	Total tested	S	I	R	VME (%)	ME (%)	mE (%)	% CA
Ampicillin	10	7	0	3	0 (0.0)	0 (0.0)	0 (0.0)	100.0
Clindamycin	66	46	0	20	5 (25.0)	2 (4.3)	1 (1.5)	87.9
Erythromycin	66	42	0	24	0 (0.0)	3 (7.1)	2 (3.0)	92.4
Fusidic acid	66	52	0	14	2 (14.3)	0 (0.0)	1 (1.5)	95.5
Gentamicin	66	55	0	11	2 (18.2)	0 (0.0)	0 (0.0)	97.0
Linezolid	76	76	0	0	0 (0.0)	0 (0.0)	0 (0.0)	100.0
Rifampin	66	64	1	1	0 (0.0)	0 (0.0)	3 (4.5)	95.0
Vancomycin	10	10	0	0	0 (0.0)	0 (0.0)	0 (0.0)	100.0
Cefoxitin screen	37	35	0	2	1 (50.0)	0 (0.0)	0 (0.0)	97.0
Inducible clindamycin resistance	10	6	0	4	4 (100)	0 (0.0)	0 (0.0)	60.0
Overall	473	393	1	79	14 (17.7)	5 (1.3)	7 (1.5)	94.5

a S, susceptible; I, intermediate; R, resistant; VME, very major error; ME, major error; mE, minor error; CA, categorical agreement. Percentage VME and ME were calculated based on the number of resistant and susceptible isolates, respectively.

Of the 1,209 (72.3%) Gram-negative AST measurements, discrepant results obtained were 17 VME, 13 ME, and 20 mE, giving an overall agreement rate for Gram-negative AST measurements of 95.9% (Table 2). The AST results where discrepancies were encountered for Gram-positive bacteria include amikacin, ceftazidime, ciprofloxacin, gentamicin, imipenem, meropenem, piperacillin-tazobactam, and trimethoprim-sulfamethoxazole, as well as testing for presence of extended spectrum b-lactamase-producing (ESBL) (Table 2, Table S5).

**TABLE 2:** dRAST versus reference method for Gram-negative bacteria from prospective clinical blood culture samples^*a*^

Antibiotic	Total tested	S	I	R	VME (%)	ME (%)	mE (%)	% CA
Amikacin	124	121	3	0	0 (0.0)	2 (1.7)	3 (2.4)	96.0
Cefotaxime	119	104	1	14	0 (0.0)	0 (0.0)	0 (0.0)	100.0
Ceftazidime	122	106	1	15	0 (0.0)	3 (2.8)	4 (3.3)	94.3
Ciprofloxacin	124	95	6	23	1 (4.3)	1 (1.1)	7 (5.6)	92.7
Gentamicin	124	116	1	7	1 (14.3)	0 (0.0)	3 (2.4)	96.8
Imipenem	120	118	2	0	0 (0.0)	1 (0.8)	3 (2.5)	97.5
Meropenem	124	123	1	0	0 (0.0)	1 (0.8)	1 (0.8)	98.4
Piperacillin/Tazobactam	122	104	1	17	12 (70.6)	2 (1.9)	3 (2.5)	86.1
Trimethoprim/Sulfamethoxazole	121	90	0	31	0 (0.0)	0 (0.0)	1 (0.8)	99.2
ESBL	109	94	0	15	2 (13.3)	0 (0.0)	0 (0.0)	98.2
Overall	1209	1071	16	122	16 (13.1)	10 (0.9)	25 (2.1)	95.9

a S, susceptible; I, intermediate; R, resistant; ESBL, extended spectrum β-lactamase-producing; VME, very major error; ME, major error; mE, minor error; CA, categorical agreement. Percentage VME and ME were calculated based on the number of resistant and susceptible isolates, respectively.

### Performance of dRAST on MDR bacteria isolates.

A total of 31 MDR bacterial isolates were inoculated and incubated as simulated blood cultures for testing with the dRAST (Table 3), and the results obtained were compared to available reference method results. All disk diffusion measurements of the isolates used in the present study were in agreement with broth microdilution data where available.

**TABLE 3:** Clinical isolates of MDR bacteria selected for testing with the dRAST^*a*^

Species	*n*
Gram positive	10
Methicillin-resistant Staphylococcus aureus	10
Gram negative	21
Acinetobacter *baumanii*	2
Escherichia coli (all ESBL)	5
Klebsiella pneumoniae	12
ESBL	8
CARBA	1
CARBA + ESBL	3
Pseudomonas aeruginosa	2
Total	31

a ESBL, extended spectrum β-lactamase-producing; CARBA, carbapenem-resistant.

Ten isolates of methicillin-resistant S. aureus (MRSA) were tested on the dRAST, resulting in 70 overall measurements that were compared between the dRAST with the disk diffusion method. The dRAST had an overall 92.9% agreement rate with disk diffusion; however, seven errors were obtained on the Gram-positive AST panel including a failure to detect one MRSA by cefoxitin screen. Of the remaining four errors, two were VMEs obtained for clindamycin, one ME obtained for gentamicin, one ME obtained for rifampin, and two errors for inducible clindamycin resistance (Table 4, Table S6).

**TABLE 4:** dRAST versus reference method for simulated blood culture samples with MRSA isolates^*a*^

Antibiotic	Total tested	S	R	VME (%)	ME (%)	mE (%)	% CA
Clindamycin	10	7	3	2 (66.7)	0 (0.0)	0 (0.0)	80.0
Erythromycin	10	6	4	0 (0.0)	0 (0.0)	0 (0.0)	100.0
Fusidic acid	10	8	2	0 (0.0)	0 (0.0)	0 (0.0)	100.0
Gentamicin	10	7	3	0 (0.0)	1 (14.3)	0 (0.0)	90.0
Linezolid	10	10	0	0 (0.0)	0 (0.0)	0 (0.0)	100.0
Rifampin	10	9	1	0 (0.0)	0 (0.0)	1 (10.0)	90.0
Cefoxitin screen	10	0	10	1 (10.0)	0 (0.0)	0 (0.0)	90.0
Overall	70	47	23	3 (13.0)	1 (2.1)	1 (1.4)	92.9

*^a^*S, susceptible; R, resistant; VME, very major error; ME, major error; mE, minor error. Percentage VME and ME were calculated based on the number of resistant and susceptible isolates, respectively.

For the 24 Gram-negative isolates tested with the Gram-negative AST panel on the dRAST, the dRAST had an overall agreement rate of 85.8% with disk diffusion based on 196 measurements on Gram-negative isolates, and obtained 27 (14.2%) discrepancies on the Gram-negative AST panel, and ESBL status for six K. pneumoniae isolates could not be detected by the dRAST (Table 5, Table S7).

**TABLE 5:** dRAST versus reference method for simulated blood culture samples with MDR Gram-negative isolates^*a*^

Antibiotic	Total tested	S	I	R	VME (%)	ME (%)	mE (%)	% CA
Amikacin	21	15	2	4	0 (0.0)	3 (20.0)	2 (9.5)	76.2
Cefotaxime	17	1	0	16	0 (0.0)	0 (0.0)	0 (0.0)	100.0
Ceftazidime	19	2	0	17	1 (5.9)	1 (50.0)	0 (0.0)	89.5
Ciprofloxacin	21	6	2	13	0 (0.0)	0 (0.0)	2 (9.5)	90.0
Gentamicin	21	14	0	7	0 (0.0)	1 (7.1)	0 (0.0)	95.0
Imipenem	21	14	4	3	0 (0.0)	0 (0.0)	3 (14.3)	86.0
Meropenem	21	13	3	5	0 (0.0)	0 (0.0)	6 (28.6)	71.0
Piperacillin/Tazobactam	19	3	3	13	3 (23.1)	0 (0.0)	2 (10.5)	74.0
Trimethoprim/Sulfamethoxazole	19	7	0	12	0 (0.0)	0 (0.0)	3 (15.8)	84.0
ESBL	17	1	0	16	0^*b*^ (0.0)	0 (0.0)	0 (0.0)	64.7^*b*^
Overall	196	76	14	106	4 (3.8)	5 (6.6)	18 (9.2)	85.8

*^a^*S, susceptible; I, intermediate; R, resistant; ESBL, extended spectrum β-lactamase-producing; VME, very major error; ME, major error; mE, minor error. Percentage VME and ME were calculated based on the number of resistant and susceptible isolates, respectively.

b ESBL not detected (ND) in dRAST for six samples.

## DISCUSSION

Conventional AST methods require manual procedures and can take 24 h to be completed from positive blood cultures, and are thus too delayed to achieve targeted treatment in the initial stages of a BSI. Hence, there is a pressing need for the development of new technology that is capable of providing a timely AST result, ideally within the working day, while being easy to set up and automated so as to reduce manual work (14). The dRAST system aims to fulfill that by providing AST results direct from positive blood cultures within 6 h with minimal initial input from the operator.

Previous studies evaluating the dRAST against various reference methods showed that it generally has a high agreement rate above 90% and low error rate when compared with various reference AST methods (7, 8, 10). The present study on the performance of dRAST showed that it had a 95.5% overall agreement with AST results obtained by disk diffusion when using prospective clinical blood culture samples. On Gram-positive prospective samples, the dRAST obtained an agreement rate with disk diffusion AST results of 95.4%. Previously published dRAST studies focusing on its performance on Gram-positive bacteria report a lower error rate of 0–4.5% in comparison to other methods such as the MicroScan Walkaway and broth microdilution. However, it was noted that these earlier dRAST studies did not contain data regarding cefoxitin screen (8, 9). In the present study, the dRAST achieved a 100% agreement with disk diffusion for cefoxitin screen on prospective clinical blood culture samples. The dRAST also performed well with Gram-negative bacteria from prospective clinical samples, achieving an agreement of 95.9% with disk diffusion.

However, the present study also observed that the overall error rates, in particular for VME and ME, obtained by the dRAST in comparison to disk diffusion were high when prospective clinical samples were analyzed. Overall error rates observed were 13.1–17.7%, 0.9–1.3%, and 1.5–2.1%, for VME, ME, and mE, respectively. Error rates for the AST of some antibiotics individually were also observed to be high. The highest VMEs observed included 100% for detection of inducible clindamycin resistance where all four resistant clinical isolates were not detected, followed by 70.6% for piperacillin-tazobactam. For ME, the highest was observed with erythromycin at 7.1%. The high VME rates could be due to the overall low number of resistant isolates encountered in the present study, and there were noticeable problems with certain antibiotic-bacteria combinations. Piperacillin-tazobactam AST in particular was most problematic and gave the highest number of VMEs, most of which occurred with E. coli but also with Klebsiella spp. This is not completely surprising following emerging evidence of performance and consistency issues with piperacillin-tazobactam AST (15). Previous studies using clinical isolates or samples with the dRAST have shown a low overall VME, ME, and mE rate; however, it seems that the total number of isolates were considered for error rate calculation (7, 10, 11). The present study used only resistant and susceptible isolates as the denominator for calculating percentage VME and ME, respectively. This makes it difficult to compare the error rates obtained between the present study and previous studies.

As Sweden has a generally low rate of MDR bacteria (13), the present study used MDR isolates to generate simulated blood culture samples for testing on the dRAST. The present study showed that the dRAST had 87.2% agreement with available disk diffusion results for the MDR bacteria isolates tested, which was lower than that obtained with prospective clinical samples with mostly susceptible isolates. On the MRSA isolates, the dRAST achieved a 92.2% on MRSA isolates. Errors were found for clindamycin (2 VMEs), gentamicin (1 ME), rifampin (1 mE), one missed isolate for the cefoxitin screen, and two missed isolates (out of 10 MRSA isolates) for inducible clindamycin resistance. The dRAST did not perform as well with MDR Gram-negative isolates, achieving an 87.6% agreement with disk diffusion results, with most of the errors obtained classified as mEs. Of the other errors, three of four VMEs came from piperacillin-tazobactam, again reflecting previously highlighted consistency issues (15). Our data obtained using MDR isolates in simulated samples suggests that the performance of the dRAST might be slightly lower with such isolates; however, the data presented here are based on a limited number of resistant isolates, and this remains to be tested rigorously in a clinical setting that encounters higher frequencies of MDR isolates.

The present study is unique in the use of an algorithm that could potentially be applied to the clinical routine by trained clinical staff to prescreen positive blood culture bottles that would be suitable for running on the dRAST. The algorithm criteria endeavored to avoid positive blood culture bottles that were polymicrobial, containing skin flora contaminants, anaerobic, or containing Streptococci based on Gram stain and morphology. The algorithm performed well and resulted in 82.6% of selected clinical blood culture samples being compatible with the dRAST. It is not logical to use the dRAST on all positive blood culture samples if there are known species that are incompatible with the system, or if there is a high blood culture contamination rate. We have previously shown that the contamination rate could be as high as 50% of all positive blood culture samples (16). Using a sample screening algorithm is thus important if the AST device should not be used for all samples, to minimize unnecessary use of dRAST consumables.

The rapid identification of microorganisms by a reliable external system avoids the problem of invalid AST tests due to microorganism identification issues that were previously observed with the Accelerate Pheno system (17). When coupled with the use of rapid identification methods such as short-term culture followed by MALDI-TOF MS, the identity of the microorganism could be input into the dRAST by early afternoon, and an AST result obtained before the end of the work shift. This was done manually in the present study, but as the dRAST has the capability to connect to the Laboratory Information System (LIS), a more streamlined workflow would involve the automatic retrieval of the bacteria identity by the dRAST from the LIS. The workflow may also be improved in future versions of the dRAST. Already, the dRAST has been further improved to be equipped with an integrated expert system (11). Future generations of the dRAST could allow an overnight option where a blood culture sample that turns positive toward the end of the shift could be run overnight on both Gram-positive and Gram-negative AST panels and the pathogen identification provided later on during the morning shift (verbal communication with Quantamatrix Inc.). Rapid ASTs are thus complementary to the clinical routine for the time being and can be costly. However, if the dRAST can be improved to take all positive blood cultures that are not contaminants, it will be of great value as the dRAST is easy to use and is able to offer more AST for a wider range of microorganisms compared to the labor-intensive EUCAST rapid AST method.

Limitations of the present study include having few isolates of some species that are not encountered frequently in our clinic, such as Citrobacter spp., Serratia marcescens, and Acinetobacter spp., and that only categorical agreement could be assessed here since the primary method used in the clinical routine is disk diffusion. The dRAST is capable of giving MIC data, but this could not be assessed as we did not use broth microdilution results since they are not performed for the vast majority of isolates in the clinical setting. Another limitation was that the exact number of blood culture bottles excluded with the sample algorithm could not be determined. Lastly, the data obtained from running prospective clinical samples reflect the performance of the dRAST in a setting that encounters mostly susceptible bacteria due to generally low antibiotic resistance levels in Sweden (13).

In conclusion, the present study demonstrated that the dRAST is promising, given that it was able to obtain >90% categorical agreement with disk diffusion on prospective clinical samples. However, we observed a high proportion of VME and ME compared to disk diffusion as the reference method that could be related to small sample sizes. Future studies with a larger sample size of isolates with varying phenotypes, and also with other reference methods that also provide MIC data so as to assess essential agreement, are warranted. The method is user friendly and fast, and it can be implemented easily in the clinical routine. The use of an algorithm might help to exclude contaminants and nonpanel microorganisms increasing the cost-effectiveness of the method.

## MATERIALS AND METHODS

### Laboratory setting, blood culture bottles, and blood culture system.

Clinical blood culture samples collected for 13 weeks between November 2019 and March 2020 by standard protocols at Karolinska University Hospital (Huddinge, Sweden) in BacT/Alert-FA Plus and BacT/Alert-FN Plus blood culture bottles (bioMérieux, Marcy-l'Étoile, France) were used for the study. All blood culture bottles that were received at the Clinical Microbiology Department, Karolinska University Hospital (Huddinge, Sweden), were incubated in the automated BacT/Alert 3D blood culture system (bioMérieux, Marcy-l'Étoile, France) until positivity, or for a maximum of 5 days.

### Sample selection algorithm for clinical blood cultures.

**FIG 3:**
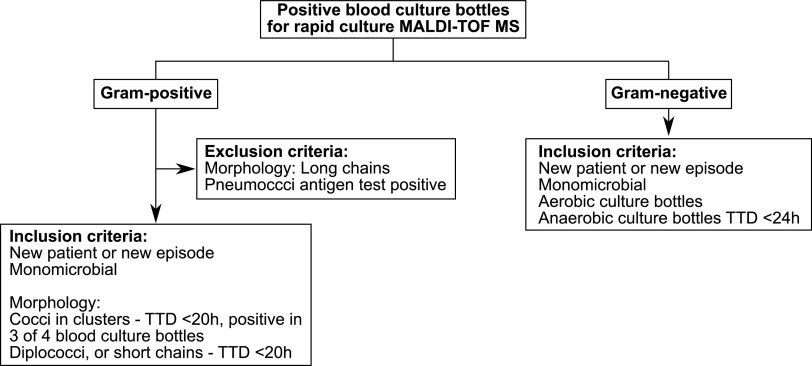
Sample selection algorithm for the dRAST. Using the algorithm, positive blood culture bottles were assessed for their suitability for analysis on the dRAST using their Gram stain and morphology. Samples that fit the inclusion criteria were analyzed on the dRAST. ID, identification; TTD, time to detection.

The number of unique patient bottles marked for rapid culture MALDI-TOF MS, and number of streptococci and CoNS isolates received, were calculated from various clinic records (Table S1). In order to simulate how the dRAST system may be used in the established clinical routine at the Clinical Microbiology Department, Karolinska University Hospital (Huddinge, Sweden), a sample selection algorithm was developed and used by clinic staff to identify blood culture samples that would be most clinically relevant for rapid AST. As the laboratory is closed at 6.00 p.m., blood bottles that signaled positive in the blood culture system between 5.00 p.m. the previous evening and 9.00 a.m. in the morning were included in the study. The algorithm aims to exclude blood culture contaminants and isolates that are not compatible with the dRAST system. We used mainly Gram stain result, number of positive blood culture bottles from the same patient, and time to positivity to identify relevant clinical positive blood culture samples for testing with the dRAST system. Only one blood culture bottle per unique BSI episode was included. Two or more episodes from the same patient could be included if they occurred more than 72 h apart. Only blood culture bottles with monomicrobial growth assessed by Gram-staining were included. In order to avoid testing contaminants, Gram-positive cocci in clusters were included when the patient had three of four blood culture bottles positive with the same bacteria or TTD was <20 h. Gram-positive blood cultures were excluded if there were cocci in long chains or if the sample had a corresponding positive pneumococci antigen test (Wellcogen Streptococcus pneumoniae Rapid Latex Agglutination Test, Thermo Scientific, Massachusetts, United States) since these bacteria are not included in dRAST system panel. Gram-negative blood cultures were included if they were in aerobic culture bottles, or if they were anaerobic culture bottles with a TTD <24 h (Fig. 3).

### Rapid pathogen identification by MALDI-TOF.

Short-term culture followed by identification with MALDI-TOF MS was used to rapidly identify pathogens in blood culture samples (18) as per standard clinical protocols. Briefly, positive blood culture bottles were removed from the culture system and 10 drops of blood culture broth were inoculated on blood agar. The agar plates were then incubated for 2 h at 37°C in 5% CO_2_ atmosphere. If no growth was visible after 2 h, the agar plates were incubated for a total of 4 h. Resulting growth was spotted in duplicates to a steel MALDI-TOF MS target plate, and 1 μL of α-Cyano-4-hydroxycinnamic acid (HCCA) matrix was added to each sample spot, followed by analysis with MALDI-TOF MS. MALDI-TOF MS scores ≥ 1.70 and ≥ 2.00 were accepted as successful identifications at genus and species level, respectively.

### Simulated blood culture samples.

Simulated blood culture samples were done using selected multidrug resistant clinical isolates that were stored in −80°C. These isolates were thawed and cultured overnight on blood agar plates at 37°C. Resulting pure colonies were suspended in 0.9% NaCl until a turbidity of 0.5 McFarland (1.5 × 10^8^ CFU/mL) before being diluted to a final 15,000 CFU/mL, from which 70 μL (1,000 CFU) was mixed with 5 mL of sterile human blood from healthy donors (Transfusion Medicine, Karolinska University Hospital, Huddinge) and inoculated into a BacT/Alert-FA Plus bottle (bioMérieux, Marcy-l'Étoile, France). The inoculated bottles were then incubated in the BacT/Alert 3D system (bioMérieux, Marcy-l'Étoile, France) and were removed after signaling positive. The bacterial suspension was also cultured on two blood agar plates as an inoculation CFU control, and CFU controls showed that the inoculum had 13 ± 5 CFU/μL. In addition, negative control bottles inoculated with the human blood without bacteria were incubated in the same way, and these were automatically discarded by the BacT/Alert 3D system (bioMérieux, Marcy-l'Étoile, France) after 5 days of incubation without detected microbial growth.

### Description and usage of the dRAST system.

The dRAST (Quantamatrix Inc., Seoul, Republic of Korea) is a system that allows rapid AST testing directly on positive blood cultures, using time-lapse microscopy and automatic microscopic image analysis to determine the growth of bacteria over time in the presence of antimicrobials. The dRAST version 2.5 (software version 1.2.5) instrument was used for the present study alongside the provided AST panels specific for either Gram-positive (QMAC-dRAST GP E19) or Gram-negative bacteria (QMAC-dRAST GN E19) specified for EUCAST recommendations. The dRAST was used as follows to simulate utilization of the instrument in clinical routine. Each weekday morning during the study period, the aforementioned sample selection algorithm was applied to blood culture samples that had turned positive overnight, and these selected samples were then prepared for the dRAST. Sample preparation involved retrieving 1 mL of suspension from the positive blood culture bottle with a sterile syringe and transfer into 5 mL polystyrene tubes (Falcon round-bottom polystyrene tubes without cap, Corning Inc.) without additional processing. Sample tubes were then inserted into the designated sample slot along with the prepackaged dRAST consumables as prompted by the dRAST instrument. The appropriate number of Gram-positive or Gram-negative AST panels were then inserted into the instrument side panel. When the test is started, the dRAST instrument automatically prepares, incubates, acquires time lapse images, and interprets AST results for each sample. As the patient samples were decoded, and the dRAST instrument used was not connected to the LIS, the dRAST was unable to retrieve the pathogen identity automatically. The pathogen identity obtained by short-term culture followed by MALDI-TOF MS was instead manually keyed into the dRAST software by 1 p.m. while the test was still running.

### Reference methods.

EUCAST standardized disk diffusion testing was used as the primary reference method (4, 12). Escherichia coli and Klebsiella pneumoniae isolates resistant to cefotaxime and/or ceftazidime were further tested for ESBL (discs from Mast Diagnostics [Merseyside, UK]) and AmpC (MIC Test Strip [Liofilchem, Roseto degli Abruzzi, Italy]). Isolates that were resistant to either or both of the cephalosporins but tested phenotypically negative for ESBL and *AmpC*, and isolates with a meropenem zone < 28 mm with the reference method (the carbapenemase screening breakpoint of EUCAST) were sent to the Public Health Agency of Sweden for further characterization of resistance mechanisms using whole genome sequencing. All Staphylococcus aureus isolates were screened for methicillin resistance with EUCAST standard disk diffusion using cefoxitin (4, 12). The presence of *mecA* or *mecC* in screen-positive isolates was confirmed using standard molecular methods (as described in reference 19).

### Data analysis.

Data for susceptible/susceptible, increased exposure/resistant (S/I/R) interpretations were exported from the dRAST system. Clinical AST results (S/I/R interpretations for disk diffusion on agar) for the chosen clinical isolates were retrieved from the Laboratory Information System (LIS). In the case of detection of ESBL-producing bacteria (bacteria resistant to β-lactam antibiotics such as penicillin and extended-spectrum cephalosporins, namely, ceftazidime and cefotaxime), cefoxitin screen, and inducible clindamycin resistance, results were reported by the dRAST as either positive (POS) or negative (NEG). POS isolates are considered resistant while NEG isolates are considered as susceptible for their respective antimicrobials. No other patient information was obtained from the LIS. The results were collated alongside each other into Excel software. Discrepant results obtained with the two AST methods were categorized as minor error (mE; dRAST = S or R and reference method = I); major error (ME; dRAST = R and reference method = S); very major error (VME; dRAST = S and reference method = R). Percentage mE was calculated based on the total number of isolates tested, while ME and VME were calculated based on the number of susceptible and resistant isolates, respectively.

### Data availability.

The data presented in this study are openly available upon request.
