# Supplementary material for: Performance of dRAST on Prospective Clinical Blood Culture Samples in a Simulated Clinical Setting and on Multidrug-Resistant Bacteria
Source: Microbiol Spectr. 2022 Mar 2;10(2):e02107-21. doi: 10.1128/spectrum.02107-21 (PMC8941874; doi:10.1128/spectrum.02107-21)
Supplement: SUPPLEMENTAL FILE 1 — Supplemental material. Download SPECTRUM02107-21_Supp_1_seq7.pdf, PDF file, 0.3 MB [file spectrum02107-21_supp_1_seq7.pdf]

Supplementary Data

Performance of dRAST^{TM} on prospective clinical blood culture samples in a simulated clinical setting and on multidrug-resistant bacteria

Alicia Y. W. Wong, Alexander T. A. Johnsson, and Volkan Özenci

Table S1. Estimated number of unique patient blood culture samples during study period

	**Total no. of positive unique patient blood culture bottles**	**Average number of bottles/week**	**Samples during 13 study weeks**
Year 2018	3050	58	754
Year 2019	3200	61	793
Week 1 October 2021	69	-	897
Week 2 October 2021	66	-	858
Week 1 November 2021	67	-	871
Week 2 November 2021	76	-	988

Table S2. Numbers of S/I/R isolates by disk diffusion for prospective Gram-positive blood culture samples

	Ampicillin			Clindamycin			Erythromycin			Fusidic acid			Gentamicin			Linezolid			Rifampin			Vancomycin			Cefotoxin screen		Inducible clindamycin resistance	
**Species**	**S**	**I**	**R**	**S**	**I**	**R**	**S**	**I**	**R**	**S**	**I**	**R**	**S**	**I**	**R**	**S**	**I**	**R**	**S**	**I**	**R**	**S**	**I**	**R**	**POS**	**NEG**	**POS**	**NEG**
E. faecalis	7	0	0													7	0	0				7	0	0				
E. faecium	0	0	3													3	0	0				3	0	0				
S. aureus				30	0	5	30	0	5	35	0	0	35	0	0	35	0	0	33	1	1				1	34	3	2
S. epidermidis				9	0	13	5	0	17	13	0	9	13	0	9	22	0	0	22	0	0				1	0	1	4
S. hominis				1	0	0	1	0	0	1	0	0	1	0	0	1	0	0	1	0	0							
S. lugdunensis				1	0	0	1	0	0	1	0	0	1	0	0	1	0	0	1	0	0				0	1		
Staphylococcus spp.				5	0	2	5	0	2	2	0	5	5	0	2	7	0	0	7	0	0							
**Total**	**7**	**0**	**3**	**46**	**0**	**20**	**42**	**0**	**24**	**52**	**0**	**14**	**55**	**0**	**11**	**76**	**0**	**0**	**64**	**1**	**1**	**10**	**0**	**0**	**2**	**35**	**4**	**6**

S, susceptible; I, intermediate; R, resistant; POS, positive; NEG, negative

Table S3. Numbers of S/I/R isolates by disk diffusion for prospective Gram-negative blood culture samples

	Amikacin			Cefotaxime			Ceftazidime			Ciprofloxacin			Gentamicin			Imipenem			Meropenem			Piperacillin/Tazobactam			Trimethoprim/Sulfamethoxazole			ESBL	
**Species**	**S**	**I**	**R**	**S**	**I**	**R**	**S**	**I**	**R**	**S**	**I**	**R**	**S**	**I**	**R**	**S**	**I**	**R**	**S**	**I**	**R**	**S**	**I**	**R**	**S**	**I**	**R**	**POS**	**NEG**
Acinetobacter spp.	2	0	0							0	2	0	2	0	0	2	0	0	2	0	0				2	0	0		
C. freundii	2	0	0	2	0	0	2	0	0	2	0	0	2	0	0	2	0	0	2	0	0	2	0	0	2	0	0		
E. cloacae	4	0	0	4	0	0	4	0	0	4	0	0	4	0	0	4	0	0	4	0	0	4	0	0	4	0	0		
E. coli	85	2	0	74	1	12	74	1	12	65	4	18	82	1	4	87	0	0	87	0	0	74	0	13	60	0	27	12	75
K. oxytoca	4	0	0	4	0	0	4	0	0	4	0	0	4	0	0	4	0	0	4	0	0	2	0	2	3	0	1	0	4
K. pneumoniae	11	0	0	10	0	1	9	0	2	8	0	3	10	0	1	11	0	0	11	0	0	8	1	2	9	0	2	2	9
K. variicola	2	0	0	2	0	0	2	0	0	2	0	0	2	0	0	2	0	0	2	0	0	2	0	0	2	0	0	0	2
P. aeruginosa	3	0	0				3	0	0	3	0	0	2	0	1	3	0	0	2	1	0	3	0	0					
P. mirabilis	4	1	0	4	0	1	4	0	1	4	0	1	4	0	1	0	2	0	5	0	0	5	0	0	4	0	1	1	4
S. marcescens	2	0	0	2	0	0	2	0	0	2	0	0	2	0	0	1	0	0	2	0	0	2	0	0	2	0	0		
Salmonella spp.	2	0	0	2	0	0	2	0	0	1	0	1	2	0	0	2	0	0	2	0	0	2	0	0	2	0	0		
**Total**	**121**	**3**	**0**	**104**	**1**	**14**	**106**	**1**	**15**	**95**	**6**	**23**	**116**	**1**	**7**	**118**	**2**	**0**	**123**	**1**	**0**	**104**	**1**	**17**	**90**	**0**	**31**	**15**	**94**

ESBL, Extended spectrum β-lactamase-producing; S, susceptible; I, intermediate; R, resistant; POS, positive; NEG, negative

Table S4. Discrepant results from Gram-positive prospective clinical blood culture samples

Antibiotic	Error (n)	Strain (n)	dRAST™	Disk diffusion
Clindamycin	VME (5)	*S. aureus* (3)<br>*S. epidermidis* (2)	S<br>S	R<br>R
	ME (2)	*S. aureus* (2)	R	S
	mE (1)	*S. aureus* (1)	I	S
Erythromycin	ME (3)	*S. aureus* (3)	R	S
	mE (2)	*S. epidermidis* (1)<br>*S. aureus* (1)	I<br>I	R<br>S
Fusidic acid	VME (2)	*S. epidermidis* (1)<br>*Staphylococcus* spp. (1)	S<br>S	R<br>R
	ME (1)	*S. epidermidis* (1)	R	S
Gentamicin	VME (2)	*Staphylococcus* spp. (2)	S	R
Rifampin	mE (3)	*S. aureus* (2)<br>*S. aureus* (1)	I<br>S	S<br>I
Cefoxitin screen	VME (1)	*S. epidermidis* (1)	NEG	POS
Inducible clindamycin resistance	VME (4)	*S. epidermidis* (1)<br>*S. aureus* (3)	NEG<br>NEG	POS<br>POS

VME, very major error; ME, major error; mE, minor error

Table S5. Discrepant results for Gram-negative bacteria from prospective clinical blood culture samples

**Antibiotic**	**Error (n)**	**Strain (n)**	**dRAST^{TM}**	**Disk diffusion**
Amikacin	ME (2)	*E. coli* (2)	R	S
	mE (3)	*E. coli* (2)	S	I
		P. mirabilis (1)	S	I
Ceftazidime	ME (3)	*E. cloacae* (1)	R	S
		P. mirabilis (1)	R	S
		S. marcescens (1)	R	S
	mE (4)	*E. coli* (1)	I	S
		E. coli (1)	I	R
		K. pneumoniae (1)	I	R
		P. mirabilis (1)	I	S
Ciprofloxacin	VME (1)	*E. coli* (1)	S	R
	ME (1)	*E. coli* (1)	R	S
	mE (7)	*E. coli* (1)	R	I
		E. coli (2)	S	I
		E. coli (1)	I	R
		K. pnemoniae (1)	I	R
		E. coli (2)	I	S
Gentamicin	VME (1)	*P. mirabilis* (1)	S	R
	mE (1)	*E. coli* (1)	S	I
Imipenem	mE (3)	*P. mirabilis* (2)	R	I
		*S. marcescen*s (1)	I	S
Meropenem	ME (1)	*P. mirabilis* (1)	R	S
	mE (1)	*P. aeruginosa* (1)	S	I
Piperacillin/Tazobactam	VME (12)	*E. coli* (11)	S	R
		K. oxytoca (1)	S	R
	ME (2)	*P. mirabilis* (1)	R	S
		P. aeruginosa (1)	R	S
	mE (3)	*E. coli* (1)	I	R
		K. pneumoniae (1)	S	I
		K. pneumoniae (1)	I	R
Trimetoprim-Sulfamethoxazole	ME (1)	*E. coli* (1)	R	S
ESBL	VME (2)	*K. pneumoniae* (1)	NEG	POS
		E. coli (1)	NEG	POS

ESBL, Extended spectrum β-lactamase-producing; dRAST, result from dRAST^{TM}; DD, result from disk diffusion; S, susceptible; I, intermediate; R, resistant; POS, positive; NEG, negative

Table S6. Discrepant results for simulated blood culture samples with MRSA isolates

**Antibiotic**	**Error (n)**	**Strain (n)**	**dRAST**	**Disk diffusion**
Clindamycin	VME (2)	*S. aureus* (2)	S	R
Gentamicin	ME (1)	*S. aureus* (1)	R	S
Cefoxitin screen	VME (1)	*S. aureus* (1)	NEG	POS

dRAST, result from dRAST™; DD, result from disk diffusion; S, susceptible; I, intermediate; R, resistant; POS, positive; NEG, negative

Table S7. Discrepant results for simulated blood culture samples with MDR Gram-negative isolates

Antibiotic	Error (n)	Strain (n)	dRAST	Disk diffusion
Amikacin	ME (3)	*E. coli* (1)	R	S
		K. pneumoniae (2)	R	S
	mE (2)	*E. coli* (1)	S	I
		E. coli (1)	R	I
Ceftazidime	VME (1)	*P. aeruginosa* (1)	S	R
	ME (1)	*P. aeruginosa* (1)	R	S
Ciprofloxacin	mE (2)	*K. pneumoniae* (2)	I	R
Gentamicin	ME (1)	*K. pneumoniae* (1)	R	S
Imipenem	mE (3)	*K. pneumoniae* (1)	S	I
		K. pneumoniae (1)	I	R
		K. pneumoniae (1)	R	I
Meropenem	mE (6)	*K. pneumoniae* (1)	I	R
		K. pneumoniae (2)	I	R
		K. pneumoniae (1)	S	I
		K. pneumoniae (1)	S	I
		P. aeruginosa (1)	I	R
Piperacillin/ Tazobactam	VME (3)	*E. coli* (2)	S	R
		K. pneumoniae (1)	S	R
	mE (2)	*E. coli* (1)	I	R
		K. pneumoniae (1)	R	I
Trimethoprim/ Sulfamethoxazole	mE (3)	*A. baumannii* (2)	I	S
		K. pneumoniae (1)	I	R
ESBL	ND (6)	*K. pneumoniae* (1)	ND	POS
		K. pneumoniae (1)	ND	POS
		K. pneumoniae (3)	ND	POS*
		K. pneumoniae (1)	ND	NEG

ESBL, Extended spectrum β-lactamase-producing; dRAST, result from dRAST™; DD, result from disk diffusion; S, susceptible; I, intermediate; R, resistant; POS, positive; NEG, negative *Carbapenem-resistant
